# Supplementary material for: Research on the impact of inclusive finance on agricultural green development: Empirical analysis of China’s main grain producing areas
Source: PLoS One. 2022 Sep 30;17(9):e0274453. doi: 10.1371/journal.pone.0274453 (PMC9524628; doi:10.1371/journal.pone.0274453)
Supplement: Attachment — Submitted filename: Review.docx [file pone.0274453.s002.docx]

*The manuscript fulfills the requirements of an academic paper however a few recommendations are suggested to improve the quality of the paper.*

1. *English Proofreading should be done.*
2. *The gap in the study should be elaborated. Why does this study need to be conducted?*
3. *Compare the results of the study with the existing studies in the field.*
4. *Conclusion and policy recommendations should be separately written.*
5. *Limitations of the study should be given.*
