# Supplementary material for: Research on the impact of inclusive finance on agricultural green development: Empirical analysis of China’s main grain producing areas
Source: PLoS One. 2022 Sep 30;17(9):e0274453. doi: 10.1371/journal.pone.0274453 (PMC9524628; doi:10.1371/journal.pone.0274453)
Supplement: Attachment — Submitted filename: Problem response.docx [file pone.0274453.s003.docx]

Thanks for your recommendations and suggestions.

Reviewer #1:

1. A good introduction consists of the importance of the topic, why this research is significant, what is known in the literature and what is unknown, what is the research problem, what theoretical framework has been used, what are the main findings, and how it contributes to the literature.

Thanks for your suggestions. For the questions raised, I have revised the introduction. The introduction mainly includes the research background and significance of this paper, the research problems, the main findings, and the main contributions of this paper. The specific modifications are as follows:

The *Opinions on Promoting Green Agricultural Development through Innovation Mechanisms* issued in 2017 called for accelerating green agricultural development. The report of the 19th National Congress of the Communist Party of China raised green agricultural development as a national strategy, *National Strategic Plan for Promoting Agriculture by Quality (2018-2022)* proposes to focus on promoting agricultural green development. China's *14th Five-Year Plan* also emphasizes promoting the green transformation of agriculture. China's main grain producing areas include 13 provinces including Hebei, Inner Mongolia, Liaoning, Jilin, Heilongjiang, Jiangsu, Anhui, Jiangxi, Shandong, Henan, Hubei, Hunan, and Sichuan. The grain output of 13 provinces accounts for more than 70% of China's grain output. Improving the agricultural green development level of the 13 provinces in China’s main grain-producing areas is of great significance for promoting the harmonious development of man and nature, for ensuring the safety of China's grain quantity and quality, and for meeting consumers' growing demand for high-quality agricultural products.

Promoting agricultural green development requires strong support from inclusive finance. Agricultural green development requires the effective allocation of various production factors such as land, capital, technology, and institutions. One of the key factors is capital, which plays a role in guiding the allocation of other production factors. It is necessary to effectively connect rural finance with the national agricultural green development policy to promote agricultural green development. Inclusive finance focuses on providing financial services to various groups of society and meeting the financial needs of different groups. Due to the inclusiveness and wide coverage of inclusive finance, inclusive finance plays a very important role in promoting agricultural green development. The development of inclusive finance can better meet the financial needs of agricultural green business entities, and can provide financial services such as funding services and insurance services for promoting agricultural green development. In 2018, the *Opinions of the Central Committee of the Communist Party of China and the State Council on the Implementation of the Rural Revitalization Strategy* emphasized that the development of inclusive finance should be focused on rural areas.

Under the background of financing constraints and high consumption of chemical products in agricultural green development, it is worth in-depth research to explore whether inclusive finance is conducive to promoting green agricultural development. It is particularly necessary for the government to formulate policies and allocate inclusive financial resources in the market.

Existing studies mainly focus on the impact of inclusive finance on urban and rural income, consumption, poverty reduction, employment, entrepreneurship and innovation. However, there are few studies on the relationship between inclusive finance and agricultural green development, and the empirical analysis of the impact of inclusive finance on agricultural green development. Therefore, whether inclusive finance affects agricultural green development and to what extent it affects agricultural green development requires further research. This paper focuses on the impact of inclusive finance on agricultural green development. This paper analyzes the development of inclusive finance, the development of green agriculture, and whether inclusive finance affects agricultural green development in 13 provinces in China's main grain producing areas.

The main contributions of this paper are as follows: Based on the theoretical analysis of the impact of inclusive finance on agricultural green development, this paper empirically analyzes the specific impact of inclusive finance on agricultural green development. By constructing a scientific evaluation index system for the development of inclusive finance, and using the coefficient of variation method to calculate Index of Financial Inclusion in 13 provinces from 2011 to 2019, this paper evaluates the development level of inclusive finance in 13 provinces; by constructing an evaluation index system for agricultural green development, using the entropy weight method to calculate the Agricultural Green Development Index of 13 provinces in China from 2011 to 2019, this paper evaluates the level of agricultural green development in 13 provinces. Using static panel (mixed least squares estimation (Pooled-OLS), random effects least squares (RE-OLS), fixed effects least squares (FE-OLS)) and dynamic panel (system GMM model) estimation methods, to analyze the specific impact of inclusive finance on agricultural green development. The results show that there is a significant positive correlation between inclusive finance and agricultural green development, and inclusive finance plays an important role in promoting agricultural green development, expanding relevant research on the relationship between inclusive finance and agricultural green development. On this basis, some targeted countermeasures and suggestions are put forward, hoping to enrich the research perspectives of inclusive finance and agricultural green development and expand related research fields.

2. The purpose of the research, the research gap, and the research field significance should be clearly stated. The current state of the art should be reviewed, and critical publications must be cited. You did not explain what this gap is. The same problem is with the aim of the paper.

Thanks for your suggestions. The research gap, the purpose of the research, and the research field significance are stated in literature review. The specific modifications are as follows:

At present, foreign scholars have less research on the impact of inclusive finance on agricultural green development, and more research is concentrated in the aspects of inclusive finance on rural residents' income, consumption, reduced urban and rural gaps, and promoting industrial structure upgrades (Bruhn, 2014) [13]. Kinnon (1973) proposes financial suppression in developing countries, and the generation of inclusive finance can effectively alleviate this phenomenon [14]. Dollar (2002) finds that rural financial development is one of the important factors to promote poverty reduction in rural areas [15]. Geoda et al. (2006) find that inclusive finance can significantly improve the income level of rural areas and lower than the median groups of income through credit, savings and other expanded financial services [16]. Lili Jiang et al. (2019) analyze the specific impact of inclusive finance on farmers' entrepreneurship[17]. Wei Zhang, et al. (2019) analyze the financial inducement mechanism to promote agricultural green development from three aspects: loan support, insurance protection and price protection [18]. Changying Hua (2022) analyzes the impact of digital inclusive finance on agricultural green development [19].

Some scholars have carried out relevant researches on inclusive finance and agricultural green development, but few literatures have studied the relationship between inclusive finance and agricultural green development. There is a lack of empirical research on the impact of inclusive finance on agricultural green development. Therefore, the main purpose of this study is to deeply analyze the internal relationship between inclusive finance and agricultural green development, to analyze the influence mechanism of inclusive finance on agricultural green development, and to conduct empirical research on the specific impact of inclusive finance on agricultural green development.

3. The novelty needs to be strengthened.

Thanks for your suggestion. The novelty of the paper are as follows:

Existing studies mainly focus on the impact of inclusive finance on urban and rural income, consumption, poverty reduction, employment, entrepreneurship and innovation. However, there are few studies on the relationship between inclusive finance and agricultural green development, and the empirical analysis of the impact of inclusive finance on agricultural green development. Based on the theoretical analysis of the impact of inclusive finance on agricultural green development, this paper empirically analyzes the specific impact of inclusive finance on agricultural green development. This paper uses both static panel and dynamic panel (system GMM model) estimation methods to empirically analyze the specific impact of inclusive financial development on agricultural green development, hoping to enrich the research perspectives of inclusive finance and agricultural green development and expand related research fields.

4. The literature review section is too long and less international paper citations. The literature review stands irrelevant the authors need to develop a conceptual and theoretical framework but a comprehensive one.

Thanks for your suggestions. I have revised the literature review. The specific modifications are as follows:

2.1 Literature review on inclusive finance

The research on the connotation, characteristics, and measurement of inclusive finance has achieved rich results. The concept of inclusive finance was put forward by The United Nations in 2005, and it is believed that inclusive finance refers to the provision of appropriate and effective financial services for all groups of society at affordable costs. Some scholars select indicators from multiple dimensions to construct an indicator system for measuring the development level of inclusive finance. For example, Beck T, et al. (2007) propose indicators to measure financial inclusion [1]; Mandira Sarma (2008) adopts three dimensions of bank penetration, financial service accessibility, and financial service use utility [2]; Arora (2010) adopts three dimensions of service scope, convenience and use cost [3]; Gupte (2012) adopts four dimensions of financial penetration, financial usability, financial transaction convenience and financial transaction cost [4]; Yaoqun Zhang, et al. (2021) adopt three dimensions of financial services geographic penetration, availability, usage of utility [5]; Wenwen Wang (2021) uses three dimensions of geographic penetration, financial product accessibility, and financial service utility [6].

2.2 Literature review on agricultural green development

The research on the connotation, characteristics, influencing factors, and measurement of agricultural green development have achieved rich results. Steven Haggblade, et al. (1989) believe that the difficulty and core of green agricultural development are the innovation of green agricultural development path and green technology innovation [7]. Unep (2011) believes that agricultural green development includes resource conservation, good ecological environment and long-term coordinated agricultural development, and has modern production methods [8]. Parviz Koohafkan, et al. (2012) believe that agricultural green development can help improve the safety and quality of agricultural products, optimize the ecological environment, and enhance the brand influence of agricultural products [9]. Adnan et al. (2019) believes that agricultural green development is affected by the individual characteristics of farmers, family characteristics, farmers' cognition, and external environmental characteristics [10]. Qi Wei, et al. (2018) evaluate China's agricultural green development level from four dimensions: resource conservation, environmental friendliness, ecological conservation and quality and efficiency [11]. Huijie Zhao, (2019) use the entropy weight method to evaluate the agricultural green development level of 13 provinces in China's main grain producing areas from four dimensions: resource conservation, environmental friendliness, high output efficiency, and life security [12].

2.3 Literature review on the impact of inclusive finance on agricultural green development

At present, scholars have less research on the impact of inclusive finance on agricultural green development，and more research is concentrated in the aspects of inclusive finance on rural residents' income, consumption, reduced urban and rural gaps, and promoting industrial structure upgrades (Bruhn, 2014) [13]. Kinnon (1973) proposes financial suppression in developing countries, and the generation of inclusive finance can effectively alleviate this phenomenon [14]. Dollar (2002) finds that rural financial development is one of the important factors to promote poverty reduction in rural areas [15]. Geoda et al.(2006) find that inclusive finance can significantly improve the income level of rural areas and lower than the median groups of income through credit, savings and other expanded financial services [16]. Lili Jiang et al. (2019) analyze the specific impact of inclusive finance on farmers' entrepreneurship[17]. Wei Zhang, et al. (2019) analyze the financial inducement mechanism to promote agricultural green development from three aspects: loan support, insurance protection and price protection [18]. Changying Hua (2022) analyzes the impact of digital inclusive finance on agricultural green development [19].

5. The conceptual framework is weak; the authors need to define a proper theoretical framework under which this study has been conducted. The theoretical background is not clear as this part's central theme of this part and why is this title not precisely stated. You need to be more clear about how theory arguments affect the constructs revealing the scientific relevance and contribution of your work.

Thanks for your suggestions. I define a proper theoretical framework in part3. The modifications are as follows:

3. Theoretical analysis of the impact of inclusive finance on agricultural green development

3.1 Financial Exclusion Theory and Inclusive Finance Theory

The concept of financial exclusion was proposed in the late 20th century. In order to chase interests to maximize interests, financial institutions have reduced or even cancel financial institutions in underdeveloped areas, making it difficult for group groups to obtain financial services. In the financial market, financial service structure is unbalanced, that is, the contradiction between the demand for financial funds and the supply of financial institutions with the supply of financial funds, which is one of the main manifestations of market failure (Kempson, Whyley,1999) [20]. Guojun Zhang et al. (2014) believe that financial exclusion provides differentiated financial services to different social populations, which will exacerbate social unfairness and make the social functions of finance cannot be realized [21].

Helms (2006) proposes to change the problem of financial exclusion in the traditional financial system, especially for customers who have been excluded by the formal financial system to obtain financial services, which can be said to be the direct purpose of developing inclusive finance [22]. Sarma & Pais (2011) believes that inclusive finance is opposite to finance, and inclusive finance means that all economic entities can enjoy financial products and services at reasonable prices fairly [23]. Jinpu Jiao (2006) believes that the inclusive financial system is based on the pursuit of sustainable and provides financial services including savings, credit, insurance and other financial services to all strata, including disadvantaged groups that are excluded from traditional financial systems outside the traditional financial system [24].

3.2 Agricultural Green Development Theory

Agricultural green development is a large agricultural in the broad sense, which is the integration and supplement of ecological agriculture, organic agriculture and natural agriculture. The relevant theories of agricultural green development can be traced back to the sustainable development theory. Both the sustainable development theory and the green growth theory originated from the Western academic circles. Gro, Harlem, Brundtland (1987) propose *sustainable developmen*t in *Our Common Future*[25]. FB Gomes, JC Moraes，DKP Neri（2001）propose *Sustainable Europe makes the world a better place: EU strategy for sustainable developmen*t [26].

3.3 Analysis of the mechanism of inclusive finance affecting agricultural green development

Inclusive finance can promote agricultural green development by improving the coverage and availability of financial services in rural areas, providing credit services, and providing insurance services.

3.3.1 Inclusive finance can promote green agricultural development by increasing the coverage and availability of financial services in rural areas

Inclusive finance can improve the coverage and availability of financial services in rural areas and reduce the cost of financial services, so it can better meet the financial needs of green agricultural business entities. Therefore, inclusive finance can promote agricultural green development. In the process of agricultural green development, there are prominent financial needs such as capital needs and insurance needs. The development of inclusive finance can effectively improve the coverage and availability of financial services by increasing the financial infrastructure such as ATM machines, bank card withdrawal points, and financial service points in rural areas, and by increasing the number of financial service personnel in rural areas. In addition, with the promotion and application of new technologies such as communications and networks, telephone banking, online banking, and e-banking have further popularized and developed in rural areas, which can further improve the coverage and availability of financial services in rural areas; and reduce the cost of providing financial services and improve the convenience and safety of providing financial services. Therefore, the development of inclusive finance can better meet the financial needs of agricultural green development.

3.3.2 Inclusive finance can promote agricultural green development by providing credit services

By providing moderate-cost credit services, inclusive finance can meet the capital needs generated in the process of agricultural green development, thereby promoting green agricultural development. On the one hand, in the process of promoting agricultural green development, it is necessary to take measures such as improving the agricultural production environment and promoting agricultural green production technology, which will generate a large demand for funds. On the other hand, green agricultural business entities have prominent capital needs in the process of promoting green agricultural development. The development of inclusive finance can better meet the capital needs generated in the process of agricultural green development. By increasing the branches or business outlets of financial institutions operating in rural areas, and by increasing the number of financial service personnel in rural areas, inclusive finance can timely understand the financial needs of different green agricultural business entities, so that financial institutions can design and develop some new, flexible credit products, which can promote agricultural green development.

3.3.3Inclusive finance can promote agricultural green development by providing insurance services

Inclusive insurance has the functions of transferring risks and dispersing risks, which is conducive to effectively dealing with natural risks, market risks and other risks in the process of agricultural green development, thereby promoting green agricultural development. In particular, agricultural insurance is conducive to dispersing and transferring the various risks faced by green agricultural business entities in the process of promoting agricultural green development. Agricultural insurance provides a guarantee for green agricultural business entities by providing compensation for losses caused by risks timely, and helps green agricultural business entities to resume production as soon as possible. Therefore, the development of inclusive insurance and agricultural insurance plays a very important role in promoting agricultural green development.

6. Please complete the information on how the results were validated. The results section is worth expanding. How do they correspond with other research on the topic?

Thanks for your suggestions. This paper uses the transformation replacement method for stable testing. The results section is expanded in part 5. The specific modifications are as follows:

5.4 Regression results of the impact of inclusive finance on agricultural green development in 13 provinces in China's main grain producing areas

From the static panel regression analysis results, it can be seen that there is a significant positive correlation between the development level of inclusive finance and agricultural green development, indicating that the development of inclusive finance has a significant role in promoting green agricultural development. The control variables such as per capita real GDP, the proportion of the added value of agriculture, forestry, animal husbandry and fishery to GDP, and the proportion of environmental protection expenditure also have a positive impact on the green development of agriculture. This shows that the increase in the actual GDP per capita, the increase in the proportion of the added value of agriculture, forestry, animal husbandry and fishery to GDP, the increase in the proportion of environmental protection expenditure, which is conducive to promoting the agricultural green development.

It can be seen from the results of dynamic panel regression analysis that after the residuals are tested, the p-values of Hansen test and Arellano-Bond in the system GMM estimation are both greater than 0.1, indicating that the model passes the correlation test, and the estimated empirical analysis result is effective. In the system GMM model, it can be seen that the development of inclusive finance has a positive impact on agricultural green development, and the control variables such as per capita real GDP and the added value of agriculture, forestry, animal husbandry and fishery to GDP also have a positive impact on agricultural green development.

Existing studies mainly focus on the impact of inclusive finance on urban and rural income, consumption, poverty reduction, employment, entrepreneurship and innovation. However, there is a lack of empirical research on the relationship between inclusive finance and agricultural green development. The results show that there is a significant positive correlation between the level of inclusive financial development, and inclusive finance plays an important role in promoting the green development of agriculture. The results of the study extends the existing studies in the field of finance and agricultural green development.

5.5 Stability test

In order to test whether the above regression analysis is stable, this paper uses the transformation replacement method for stable testing. With the development of mobile information technology and digital technology, inclusive finance gradually combines with digital technology, producing digital inclusive finance. In 2016, the G20 meeting formulated a high -level principle of digital inclusive finance, and digital inclusive finance gradually became an important part of inclusive finance. Therefore, this paper uses the Digital Inclusive Financial Index calculated by Peking University instead of the previously calculated Inclusive Financial Index for a stable test. It can be seen from the regression results of Table 7 that the impact of digital inclusive finance on agricultural green development is significantly positive in the mixed minimum daily estimation model, in the fixed effect model, in the random effect model, and in the system GMM model. The regression results are consistent with the regression results in **Table 6** above, indicating that the study of this article has good stability, and inclusive finance has a significant role in promoting agricultural green development.

Table 7 Results of stability regression analysis

| variable | Mixed Least Squares Estimation | fixed effects | random effects) | system GMM |
| --- | --- | --- | --- | --- |
| L.agi |  |  |  | 0.619***  （2.630） |
| ifi | 0.021***  (4.548) | 0.021***  (3.676) | 0.022***  (5.365) | 0.017**  （2.520） |
| lnrgdp | 0.149***  (16.932) | 0.088***  (2.853) | 0.081***  (3.838) | 0.032  （1.560） |
| is | 1.392***  (17.101) | 0.666***  (2.895) | 0.616***  (3.413) | 0.325  （1.651） |
| ef | 1.114  (3.054) | 1.502**  (3.546) | 1.323***  (3.554) | 0.102  （0.232） |
| Constant term |  | 0.166**  (2.900) | 0.184***  (4.400) | 0.058  （1.151） |
| N | 117 | 117 | 117 | 104 |
| *R*^2^ | 0.444 | 0.883 | 0.771 |  |
| Arellano-Bond |  |  |  | 0.347 |
| Hansen test |  |  |  | 0.179 |

p < 0.1, **p < 0.05, ***p < 0.01

7. The authors can also show how this study differs from other studies published in the PLOS One journal.

Thanks for your suggestions. On the basis of the theoretical analysis of the impact of inclusive finance on agricultural green development, this paper conducts empirical research on the specific impact of inclusive finance on agricultural green development, which extends the field of inclusive finance and agricultural green development in the PLOS One journal.

8. By the way, I would recommend that English writing should be proofread since there are lots of grammatical and technical errors throughout this paper.

Thanks for your recommendations. English Proofreading has been done in by an English teacher from Suqian College.

Reviewer #2:

1. English Proofreading should be done.

Thanks for your suggestion. English Proofreading has been done by an English teacher from Suqian College.

2.The gap in the study should be elaborated. Why does this study need to be conducted?

Thanks for your suggestion. The gap and the purpose of this paper are elaborated in part2. The modifications are as follows:

The gap in the existing study mainly including: some scholars have carried out relevant researches on inclusive finance and agricultural green development, but few literatures have studied the relationship between inclusive finance and agricultural green development. There is a lack of empirical research on the impact of inclusive finance on agricultural green development. Therefore, the main purpose of this study is to deeply analyze the internal relationship between inclusive finance and agricultural green development, to analyze the influence mechanism of inclusive finance on agricultural green development, and to conduct empirical research on the specific impact of inclusive finance on agricultural green development.

3.Compare the results of the study with the existing studies in the field.

Thanks for your suggestion. This paper compares the results of the study with the existing studies in the field in part 5. The modifications are as follows:

Existing studies mainly focus on the impact of inclusive finance on urban and rural income, consumption, poverty reduction, employment, entrepreneurship and innovation. There are few studies on the relationship between inclusive finance and agricultural green development, and the empirical analysis of the impact of inclusive finance on agricultural green development. The results of the study extends the existing studies in the field of finance and agricultural green development. There is a lack of empirical research on the relationship between inclusive finance and agricultural green development in the existing studies. This paper uses both static panel and dynamic panel (system GMM model) estimation methods to empirically analyze the specific impact of inclusive financial development on agricultural green development. The results show that there is a significant positive correlation between the level of inclusive financial development, and inclusive finance plays an important role in promoting the green development of agriculture.

4. Conclusions and policy recommendations should be separately written.

Thanks for your suggestion. Conclusions and policy recommendations are written separately in part 6. The modifications are as follows:

6. Conclusions and policy recommendations

6. 1 Conclusions

This paper uses both static panel and dynamic panel (system GMM model) estimation methods to empirically analyze the specific impact of inclusive financial development on agricultural green development. The results show that there is a significant positive correlation between the level of inclusive financial development, real GDP per capita, the proportion of the added value of agriculture, forestry, animal husbandry and fishery in GDP, the proportion of environmental protection expenditure and agricultural green development. Therefore, this paper draws the following conclusions: improving the development level of inclusive finance is conducive to promoting agricultural green development; the increase in per capita real GDP is conducive to promoting agricultural green development; the greater the proportion of the added value of agriculture, forestry, animal husbandry and fishery in GDP, the more conducive to promoting agricultural green development; the increase in the proportion of environmental protection expenditure is conducive to promoting agricultural green development. Based on its findings, this paper proposes the following policy recommendations.

6.2 Policy recommendations

6.2.1 Developing inclusive finance to better promote agricultural green development

Inclusive finance has a significant role in promoting agricultural green development. Therefore, it is necessary to vigorously develop inclusive finance to better promote agricultural green development. On the one hand, it is suggested to further improving the broad coverage of inclusive finance, further strengthen the construction of financial institutions' outlets, ATM machines, and cash-out points for farmers, and the construction of digital networks and other inclusive financial infrastructure in China's main grain producing areas. At the same time, increasing the number of financial practitioners in the rural areas of China's main grain producing areas can provide better financial services for promoting green agricultural development. On the other hand, it is better to further optimize inclusive financial products in China's major grain producing areas. On the basis of further understanding the types of financial needs of green agricultural operators and their characteristics, rural financial institutions can optimize the design of inclusive green credit products and inclusive green insurance products with flexible terms, flexible quotas, diverse borrowing methods, and moderate costs. Inclusive green financial products can help to better meet the capital needs and risk-transferring needs of green agricultural business entities. Therefore, agricultural green development can be promoted by providing better financial support and services.

6.2.2 Promoting economic growth to better promote agricultural green development

Economic growth plays a significant role in promoting agricultural green development. Therefore, it is necessary to vigorously promote the economic growth of China’s major grain producing areas to better promote agricultural green development. It is necessary to vigorously develop local leading industries and advantageous industries to promote economic growth according to the resource endowments and advantageous resources of each province. Economic growth can improve people's income level, increase people's demand for high-quality agricultural products, and enhance people's purchasing power for high-quality agricultural products, which is conducive to promoting agricultural green development.

6.2.3 Promoting the development of agriculture, forestry, animal husbandry and fishery to better promote agricultural green development

Agriculture, forestry, animal husbandry and fishery have a significant role in promoting agricultural green development. Therefore, it is necessary to further promote the development of agriculture, forestry, animal husbandry and fishery in China’s main grain producing areas to increase the proportion of agriculture, forestry, animal husbandry and fishery in GDP to better promote agricultural green development. In China’s main grain producing areas, the development of agriculture, forestry, animal husbandry and fishery is more important, which is not only related to food security, but also to the long-term stability of the country. Therefore, it is necessary to increase the investment in agriculture, forestry, animal husbandry and fishery in China's main grain producing areas. More attention should be paid to the green development of agriculture, forestry, animal husbandry and fishery.

6.2.4 Increasing environmental protection expenditures to better promote agricultural green development

Environmental protection expenditure has a significant role in promoting agricultural green development. Therefore, it is necessary to further increase the proportion of environmental protection expenditure in total fiscal expenditure to better promote agricultural green development. The environmental protection expenditure in China's main grain producing areas should be increased, and the increase in the proportion of environmental protection expenditure in various regions should be higher than the increase in the proportion of local financial expenditure, which is conducive to further improving the ecological environment of the main grain producing areas, optimizing the ecological environment for agricultural green development, promoting agricultural green development.

5. Limitations of the study should be given.

Thanks for your suggestion. Limitations of the study is given in part 1. The modifications are as follows:

Some limitations of this paper are as follows: This paper studies the impact of inclusive finance on agricultural green development from a macro perspective, and the relationship between the two needs to be explored from a micro perspective in the future. The data in this article belongs to the short panel, the analysis is short-term analysis, and further long-term analysis can be done in the future.
